# Gene Expression Profile of the Human Colorectal Carcinoma LoVo Cells Treated With Sporamin and Thapsigargin

**DOI:** 10.3389/fonc.2021.621462

**Published:** 2021-05-25

**Authors:** Chun Yang, Si-Jia Chen, Bo-Wen Chen, Kai-Wen Zhang, Jing-Jie Zhang, Rong Xiao, Peng-Gao Li

**Affiliations:** ^1^ School of Public Health, Capital Medical University (CMU), Beijing, China; ^2^ Beijing Key Laboratory of Environmental Toxicology, CMU, Beijing, China; ^3^ Beijing Key Laboratory of Clinical Epidemiology, CMU, Beijing, China; ^4^ National Center for Child Nutriment Quality Supervision and Testing, China National Children’s Center, Beijing, China

**Keywords:** sporamin, colorectal cancer, RNA sequencing, transcriptome, gene expression profile, thapsigargin

## Abstract

Sporamin, a proteinase inhibitor isolated from the sweet potato (*Ipomoea batatas*), has shown promising anticancer effect against colorectal cancer (CRC) *in vitro* and *in vivo* but its mechanisms of action are poorly understood. In the present study, high throughput RNA sequencing (RNA-seq) technology was applied to explore the transcriptomic changes induced by sporamin in the presence of thapsigargin (TG), a non-12-O-tetradecanolphorbol-13-acetate type cancer promoter, in the LoVo human CRC cells. Cellular total RNA was extracted from the cells after they were treated with vehicle (CTL), 1 μM of thapsigargin (TG), or 1 μM of TG plus 30 μM of sporamin (TGSP) for 24 h. The migratory capacity of the cells was determined by wound healing assay. The gene expression profiles of the cells were determined by RNA-seq on an Illumina platform. GO enrichment analysis, KEGG pathway analysis, protein-protein interaction (PPI) network construction, and transcription factors (TF) prediction were all performed based on the differentially expressed genes (DEGs) across groups with a series of bioinformatics tools. Finally, the effect and potential molecular targets of the sporamin at the transcriptome level were evaluated. Sporamin significantly inhibited the migration of cells induced by TG. Among the 17915 genes detected in RNA-seq, 46 DEGs were attributable to the effect of sporamin. RT-PCR experiment validated that the expression of RGPD2, SULT1A3, and BIVM-ERCC5 were up-regulated while NYP4R, FOXN1, PAK6, and CEACAM20 were down-regulated. Sporamin enhanced the mineral absorption pathway, worm longevity regulating pathway, and pyrimidine metabolism pathway. Two TFs (SMIM11A and ATOH8) were down-regulated by sporamin. HMOX1 (up-regulated) and NME1-NME2 (down-regulated) were the main nodes in a PPI network consisting of 16 DEGs that were modulated by sporamin in the presence of TG. Sporamin could favorably alter the gene expression profile of CRC cells, up-regulating the genes that contribute to the homeostasis of intracellular metal ions and the activities of essential enzymes and DNA damage repairment. More studies are warranted to verify its effect on specific genes and delineate the mechanism of action implicated in the process.

## Introduction

Colorectal cancer (CRC) is the world’s fourth deadliest cancer that kills approximately 700,000 people annually. Besides genetic factors, environmental factors such as an unhealthy eating habit and a sedentary lifestyle also play an important role in its etiology (1). The treatment of CRC is now hampered by many factors such as incomplete surgical resection, severe adverse effects of chemotherapy, development of drug resistance, and a lack of specificity in targeting solely tumor cells (2, 3). More importantly, tumor metastasis is almost incurable at present. Therefore, searching for novel chemopreventive agents that can suppress tumor metastasis remains a hot spot in this area.

Plants are rich sources of novel bioactive compounds that have promising anticancer activities against various cancers including CRC (4, 5). Sporamin is a serine proteinase inhibitor found in the dicotyledonous plant sweet potato (*Ipomoea batatas*) (6), and has a potent antioxidant (7), anti-obesity (8), and anticancer effect. It can suppress proliferation and induce apoptosis of many malignant cells such as *HT29*, *HCT116*, and *SW480* CRC cells (9), the *EC9706* and *EC109* human esophageal squamous cancer cells (10), the *Tca8113* human tongue carcinoma cells (11), and the *PANC-1* human pancreatic cancer cells (12). However, to date, the mechanisms of action responsible for the anticancer effect of sporamin are still poorly understood because of the limitations of the experimental technologies used in these studies. The emergence of the RNA sequencing (RNA-seq) technology has made it possible for researchers to look at the changes within the cells at the transcriptomic level and find multiple molecular targets of the experimental treatment at the same time. Therefore, to investigate the anti-metastasis effect of sporamin in CRC, in the present study, the human LoVo CRC cells were stimulated with thapsigargin (TG), a non-12-O-tetradecanolphorbol-13-acetate (TPA) type (13) cell migration promoter (14). The changes induced by sporamin treatment at the transcriptome level were examined by RNA sequencing of the cells. The results were then analyzed with a series of bioinformatics tools to elucidate the potential molecular targets of sporamin in the cells.

## Methods

### Materials

Sporamin was purified from the sweet potato, as previously described (15). The *LoVo* colon carcinoma cells were purchased from the Tumor Cell Bank of the Chinese Academy of Medical Sciences (Beijing, China). Thapsigargin (TG) and trypsin were purchased from Sigma-Aldrich^®^ (St. Louis, USA). DNase I and cDNA Reverse Transcription Kit were purchased from Thermo Scientific (MA, USA). TRIzol^®^ Reagent was from Invitrogen (MA, USA). Anhydrous ethanol, chloroform, and isopropanol were from Beijing Chemical Reagent Company (Beijing, China). Dulbecco’s Modified Eagle Medium (DMEM)-high glucose, fetal bovine serum (FBS), and phosphate balanced solution were from Corning (New York, USA). The penicillin-streptomycin mixture was from Keygen Biotech (Nanjing, China).

### Cell Culture

The *LoVo* cells were grown in DMEM supplemented with 10% FBS, 1% penicillin, and 1% streptomycin in 100 mm dishes and maintained at 37°C in a humidified atmosphere containing 5% CO_2_. The medium was changed every three days. Cells grown to confluence were serum-starved overnight and then treated with or without 1 μM of TG for 24 h. To observe the effect of sporamin on TG-treated cells, 30 μM of sporamin was dissolved in the DMEM medium in addition to the TG.

### Wound-Healing Assay

A wound-healing assay was conducted to evaluate the migratory ability of the cells. Briefly, cells were seeded onto 6-well plates and cultured to full confluence. After overnight serum-starvation, wounds of ~1 mm width were created with a pipet tip. The cells were washed once with PBS and then incubated in serum-free media containing different treatment reagents (vehicle, TG, and TG+sporamin) for 24 h, respectively. Images of the cell wounds were captured under a microscope and semi-quantitatively analyzed using the NIH *ImageJ* software (https://imagej.nih.gov). Briefly, cell migratory ability is measured based on the comparison between the remaining distances from the front-line of the cells at the edge of scratch to the middle-line of the scratch after 24 hours of different experimental treatment with the distances before treatment and the results are presented in a percentage form, the longer the remaining distance the slower the migration speed, the shorter the distance the faster the migration speed. That is, cell migration= (the distance from the cell-front to the middle line of the scratch at 0h - the distance from the cell-front to the middle line of the scratch at 24h)/the distance from the cell-front to the middle line of the scratch at 0h * 100%.

### RNA Sequencing

Nine samples [three vehicle-treated control samples (CTL), three treated with 1 μM of TG for 24 h (TG), three treated with 1 μM of TG plus 30 μM of sporamin for 24 h (TGSP)] of the cells were sent to the Beijing Genomics Institute (BGI, Guangdong, China) and sequenced as follows: (i) 1 μg of the total cellular RNA was extracted using the TRIzol^®^ reagent and treated with DNaseI at 37°C for 20 min to digest DNA. Oligo dT magnetic beads were used to select mRNA with polyA tail; (ii) the purified mRNA were fragmented and reverse transcribed to double-strand cDNA (dscDNA) by using the N6 random primer (Sequence: 5’ d(N6) 3’ [N=A, C, G, T]); (iii) the ds cDNAs were treated by a traditional process, including end repairing with phosphate at the 5′ end and stickiness ‘A’ at the 3′ end, ligation, and adaptor with stickiness ‘T’ at the 3′ end; (iv) two specific primers were used to amplify the ligation product; (v) the double-stranded polymerase chain reaction (PCR) products were heat-denatured to single-strand and circularized by splint oligo and DNA ligase; (vi) sequencing was performed on the BGISEQ-500 platform with the prepared library.

### Bioinformatics

Gene expression levels were quantified by using the RSEM software (16). The multi-dimensional scaling (MDS) plot of the RNA samples was drawn using the plot MDS function in edgeR. The distance represented the leading log-fold-changes between each pair of RNA samples, the average (root-mean-square) of the largest absolute log-fold changes (17). This visualizes the differences between the expression profiles of different samples in two dimensions. DEGseq (18) was adopted to screen the differentially expressed genes (DEGs) among the CTL, TG, and TGSP groups. The criteria for the selection of the DEGs were $\left|\log_{2}\;fold\;change\right|\geq 1$ and Q-value ≤ 0.001. The WEGO software (19) was used to conduct Gene Ontology (GO) functional classification and annotation for screened DEGs. Pathway enrichment analysis of DEGs was performed based on the KEGG (Kyoto Encyclopedia of Genes and Genomes) database (20) using the phyper algorithm in R (v3.4.1). The Cluster software (21, 22) obtained the gene expression clustering results using the Euclidean distance matrix as the matrix formula and displayed with the java Treeview (21). Mfuzz (v 2.34.0) was used to do soft clustering and adjust the result (22). The heat map was constructed using the heatmap package in R. To predict the transcription factors (TF) from the DEGs, get orf (EMBOSS 6.5.7.0. http://www.bioinformatics.nl/cgi-bin/emboss/help/getorf) was used to find the open reading frame (ORF) of each DEG, which was then aligned with the animal TF database (v2.0. http://www.bioguo.org/AnimalTFDB) using DIAMOND (v0.8.31. https://github.com/bbuchfink/diamond) (23). To obtain the interactions between DEGs-encoded proteins, the STRING Database (v10. http://string-db.org/) was used to map the DEGs directly to the interaction (24) and Cytoscape (https://cytoscape.org/) was used to visualize the interaction networks (25).

### RNA Isolation and Reverse-Transcription Quantitative Polymerase Chain Reaction (RT-qPCR)

Total RNA from cells was extracted with TRIzol reagent according to the manufacturer’s instructions. Complementary DNA was generated by using the PrimeScript™ RT reagent kit. RT-qPCR was performed by using the SYBR^®^ Premix Ex Taq™ II kit. All reactions were performed in triplicate under the following procedures: 95°C for 10 min, followed by 37 cycles of 95°C for 15 seconds, 60°C for 30 seconds, and 72°C for 30 seconds. Glyceraldehyde-3-phosphate dehydrogenase (GAPDH) was used as the housekeeping gene. The 2*^-ΔΔCq^* method was used for the calculation of the relative gene expression levels (26). The primers used for RT-qPCR are listed in **Supplementary Table S1**.

## Results

### Wound-Healing Assay

**Figure 1:**
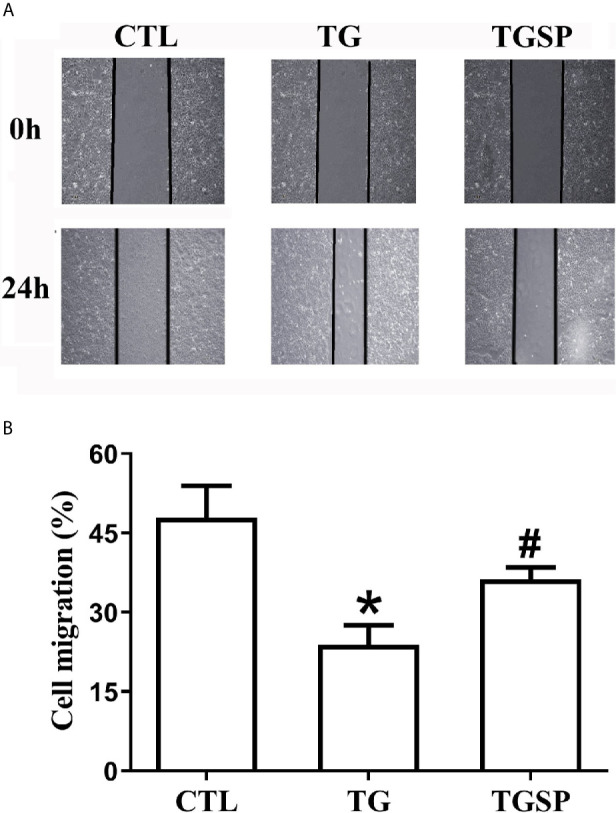
Representative Images of the wound-healing assay showing migratory ability of the LoVo cells under different treatment conditions (40×). **(A)** Wound healing assays presented the migration ability of the *LoVo* cells in CTL, TG and TGSP group. **(B)** Cell migration is measured semi-quantitatively using the NIH *ImageJ* software (https://imagej.nih.gov) where cell migration= (the distance from the cell-front to the middle line of the scratch at 0h - the distance from the cell-front to the middle line of the scratch at 24h)/the distance from the cell-front to the middle line of the scratch at 0h * 100%. *P < 0.05 *vs*. CTL, ^#^P < 0.05 *vs*. TG.

Images of the wound healing assay in **Figure 1A** show that, compared to the CTL group, the migratory capacity of cells stimulated with TG was significantly increased. In contrast, the migration of cells was suppressed by simultaneous sporamin treatment as indicated by a slower healing speed of the wound in the TGSP group. Semi-quantitative measurement of the cell migration with the *ImageJ* software in **Figure 1B** reveals that the migratory ability of the cells in the CTL, TG and TGSP groups were 47.87% ± 6.07%, 23.84% ± 3.66%, and 36.22% ± 2.27%, respectively (F=9.29, P=0.00).

### RNA-Seq Data Quality

**Figure 2:**
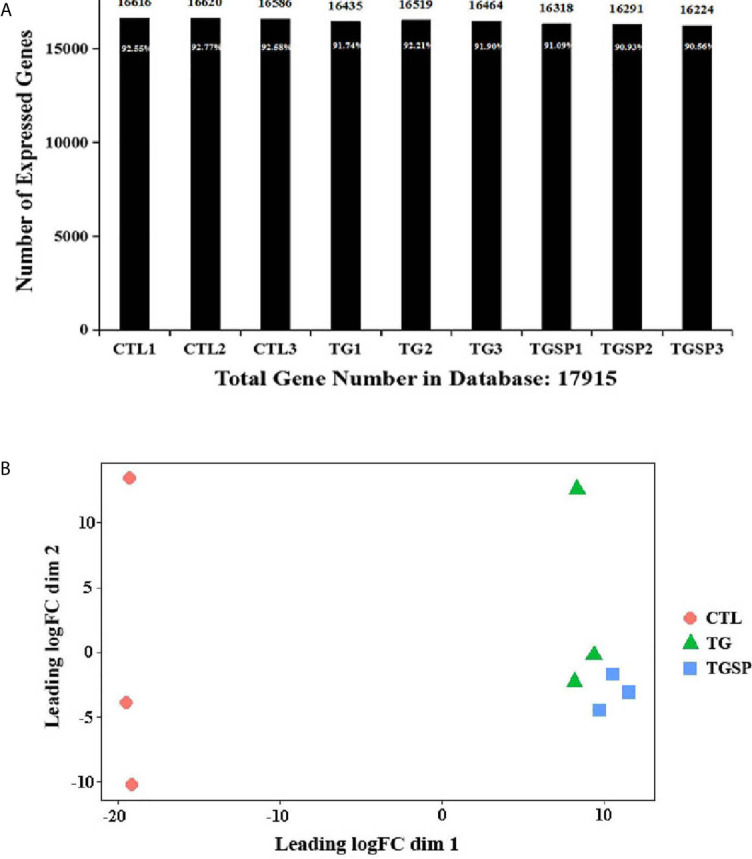
RNA sequencing data. **(A)** The number of identified genes. The X-axis indicates the sample name. Y-axis indicates the number of identified expressed genes. The proportion at the top of each bar equals the expressed gene number divided by the total gene number reported in the database. **(B)** Multi-dimensional scaling (MDS) plot assessing the similarities/differences of the expression profiles shows a clear separation between CTL, TG and TGSP group.

The sequencing generated, on average, 23,941,067 raw sequencing reads and 23,923,152 clean reads after filtering out low-quality reads. The numbers of the reads and their quality metrics for each sample are listed in **Supplementary Table S2**. The average mapping ratio with the reference genome is 60.76%. The average mapping ratio with the reference genes is 36.18%. A total of 17,915 genes were detected. RSEM-quantified gene expression levels and the numbers of identified expressed genes in each sample are shown in **Figure 2A**. A multi-dimensional scaling (MDS) plot assessing the similarities/differences among the expression profiles of the samples shows a clear separation between the CTL, TG, and TGSP samples (**Figure 2B**).

### Differently Expressed Genes (DEGs)

**Figure 3:**
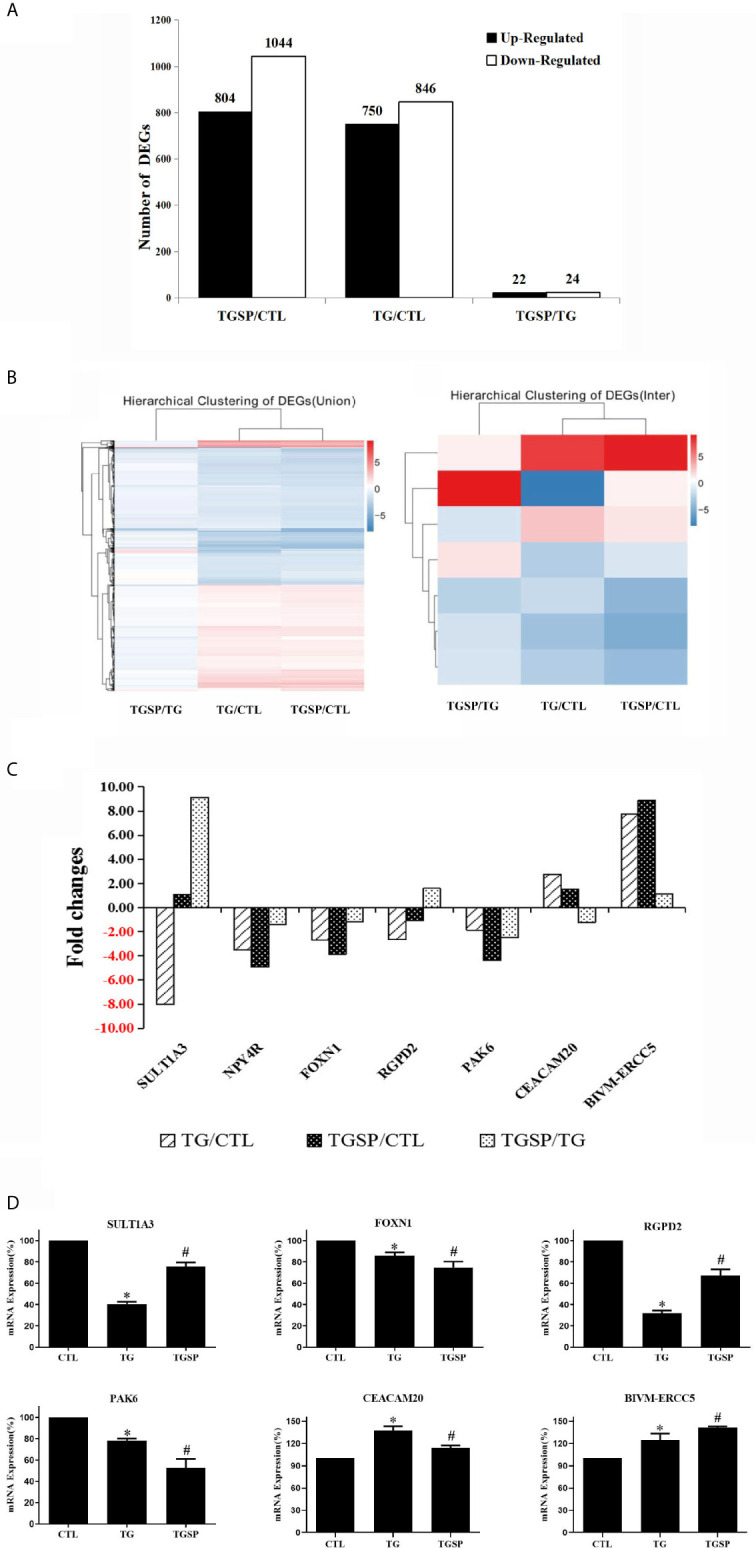
The DEGs and RT-qPCR validation experiment. **(A)** Summary of the number of the up- and down-regulated DEGs obtained from every comparison pairs. **(B)** Hierarchical clustering analysis of all of the DEGs (left panel) and the seven commonly shared DEGs across the three groups (right panel). Expression data are presented as a data matrix where each row represents a gene, and each column represents a comparison of two groups. Expression levels are shown according to the color scale, and red or blue indicate the expression levels above or below the median, respectively. **(C)** Fold changes of the seven commonly shared DEGs across the three comparison pairs. **(D)** RT-qPCR validation assay of the selected DEGs after the different experimental treatment. *P < 0.05 *vs*. CTL, ^#^P < 0.05 *vs*. TG.

As shown in **Figure 3A**, there are 1596 DEGs between the TG and control group, 1848 DEGs between the TGSP and control group, 46 DEGs between TGSP and TG group under the criteria of $\left|\log_{2}\;fold\;change\right|\geq 1$, and a Q-value < 0.001. Compared to the control group, 750 genes were significantly up-regulated, and 846 were significantly down-regulated in the TG group. More DEGs were found when sporamin was given along with TG, where 804 genes were significantly up-regulated, and 1044 genes were significantly down-regulated. When comparing the TGSP to the TG group, 22 genes were significantly up-regulated, and 24 were significantly down-regulated, suggesting that these differences could be attributed to sporamin treatment. The top 20 up- and down-regulated DEGs in each comparison pair are listed in **Supplementary Table S3** through **Supplementary Table S8**. The hierarchically clustered heatmaps in **Figure 3B** displays that the seven DEGs’ expression patterns were quite different in the TGSP group than the TG group. **Figure 3C** demonstrated that five of the DEGs were down-regulated, and two genes were up-regulated in the TG group in comparison with the CTL group. Among the five down-regulated genes, three were further down-regulated by simultaneous sporamin treatment, i.e., *NPY4R, FOXN1*, and *PAK6* (from -3.52-fold to -4.91-fold, from -2.69-fold to -3.89-fold, and from -1.86-fold to -4.35-fold, respectively), suggesting that sporamin had enhanced the effect of TG on these genes. As a result, the down-regulating effect of sporamin on *NPY4R, FOXN1*, and *PAK6* was -1.39-fold, -1.20-fold, and -2.49-fold stronger than TG, respectively. The regulation of TG on the remaining two genes, i.e., *SULT1A3* and *RGPD2*, were antagonized by simultaneous sporamin treatment (from -8.01-fold to 1.09-fold, and from -2.66 -fold to -1.06 -fold, respectively), indicating that sporamin had an opposite effect against TG on these two genes. For the two genes that were up-regulated by TG versus the control, the expression of *CEACAM20* was up-regulated 2.76-fold by TG but only 1.53-fold by TGSP, implying that sporamin had down-regulated the expression of *CEACAM20* by -1.22-fold. Compared to the control, the expression of *BIVM-ERCC5* was up-regulated 7.76-fold by TG and 8.88-fold by TGSP, implying that sporamin had enhanced TG’s up-regulation effect on *BIVM-ERCC5* by 1.12-fold. Taken together, it could be concluded that sporamin had up-regulated expression of *SULT1A3*, *RGPD2*, and *BIVM-ERCC5* but down-regulated *NYP4R, FOXN1, PAK6*, and *CEACAM20* in the presence of TG in *LoVo* cells. Moreover, the RT-PCR validation experiment shown in **Figure 3D** confirmed that the changes in the mRNA expression of RGPD2, SULT1A3, BIVM-ERCC5, FOXN1, PAK6, and CEACAM20 were consistent with the RNA-seq results.

### Gene Ontology (GO) Analysis

Based on the GO annotations of the DEGs after TG and sporamin treatment, we were able to have a better understanding of the effect of TG and sporamin in *LoVo* cells in terms of the three GO classifications-cellular component (CC), biological process (BP), and molecular function (MF).

#### Cellular Component (CC)

**Figure 4:**
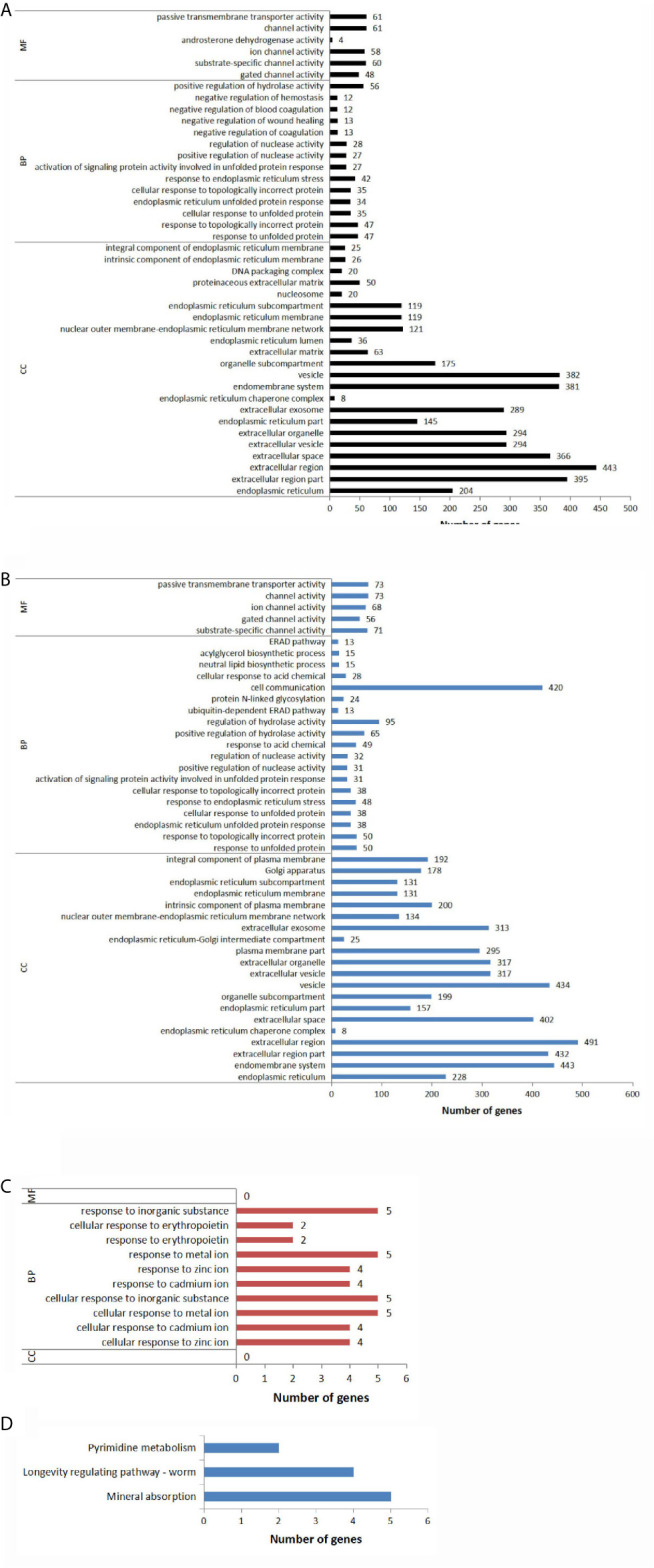
Significantly different GO terms and KEGG annotations across the groups. The expression level of the genes in the different groups and the resulting differentially expressed genes (DEGs) were subjected to the gene ontology (GO) analysis in terms of three GO classification-cellular component (CC), biological process (BP), and molecular function (MF). **(A)** Enriched GO terms with a *P-value <*0.05 for the DEGs obtained from the TG *vs*. CTL comparison pair. **(B)** Enriched GO terms with a *P-value <*0.05 for the DEGs obtained from the TGSP *vs*. CTL comparison. **(C)** Enriched GO terms with a *P-value <*0.05 for the DEGs obtained from the TGSP *vs*. TG comparison. **(D)** Significantly enriched KEGG pathways related to the DEGs obtained from the TGSP *vs*. TG comparison.

As shown in **Figure 4A** and **Supplementary Table S9**. Compared to the control group, 204 DEGs were annotated to the endoplasmic reticulum in TG group. 145 genes were annotated to endoplasmic reticulum part; 8 genes were annotated to endoplasmic reticulum chaperone complex; 36 genes were annotated to endoplasmic reticulum lumen; 119 genes were annotated to endoplasmic reticulum membrane; 119 genes were annotated to endoplasmic reticulum sub-compartment; 26 genes were annotated to an intrinsic component of endoplasmic reticulum membrane; 25 genes were annotated to an integral component of endoplasmic reticulum membrane. **Figure 4B** and **Supplementary Table S10** show that the endoplasmic reticulum was still the main CC affected by the experimental treatment in the TGSP group compared to the control group; seven CC terms that had a *P-value <*0.05 were related to the endoplasmic reticulum. **Figure 4C** and **Supplementary Table S11** show that, compared to the TG group, no CC terms with a *P-value <*0.05 were found in the TGSP group, implying that the effect of TG was overwhelming in both groups and simultaneous sporamin treatment had not changed the CC involved in response to TG exposure.

#### Biological Process (BP)

With regards to BP, **Figure 4A**, and **Supplementary Table S9** show that compared to the control group, after TG exposure, the top-seven terms with the lowest *P-values* were related to the endoplasmic reticulum stress (ERS) response such as the response to unfolded protein. Besides, 27 DEGs were related to the positive regulation of nuclease activity; 12 genes were related to the negative regulation of blood coagulation; 13 genes were related to regulation of wound healing; 56 genes were related to positive regulation of hydrolase activity. It means that these biological processes might be responsible for the tumor promotion effect of TG. **Figure 4B** and **Supplementary Table S10** show that, compared to the control group, the BP terms enriched in the TGSP group were similar to that in the TG group with few exceptions. For example, 49 DEGs were related to acid chemical; 24 genes were related to protein N-linked glycosylation; 420 genes were related to cell communication; 15 genes were related to neutral lipid biosynthetic process; 15 genes were related to acylglycerol biosynthetic process. To show the effect of sporamin more clearly, **Figure 4C** and **Supplementary Table S11** display that, in terms of the BPs, the differences between the TGSP and the TG group were all related to the cellular responses to metal ions as well as some related processes such as the response to erythropoietin and the response to the inorganic substance, implying that homeostasis of the metal ions such as zinc and the related biological processes were possibly the main target of the sporamin in the presence of TG in the *LoVo* cells.

#### Molecular Function (MF)

On analyzing MF, **Figure 4A**, and **Supplementary Table S9** show that, compared to the control group, TG exposure mainly affected the expression of the genes related to ion metabolism where 48 DEGs were significantly annotated to gated channel activity; 60 genes were annotated to substrate-specific channel activity. Moreover, 4 DEGs were associated with androsterone dehydrogenase activity; 61 genes were related to passive transmembrane transporter activity, consistent with the previous studies that TG discharges intracellular Ca^2+^ stores by specific inhibition of the endoplasmic reticulum Ca^2+^-ATPase (27). Similar findings were obtained in the TGSP group (**Figure 4B** and **Supplementary Table S10**). However, when comparing the MF terms between the TGSP and the TG group, no terms with a *P-value <*0.05 were found (**Figure 4C** and **Supplementary Table S11**). Collectively, GO analysis indicated that the effect of sporamin in TG’s presence was mainly manifested as its impact on the biological processes in the *LoVo* cells.

### KEGG Pathway Analysis

KEGG pathway analysis was performed to investigate the critical pathways associated with the present study’s DEGs. As shown in **Figure 4D**, only three pathway categories were significantly (*P<0.05*) enriched for the 11 DEGs found between the TGSP and the TG group. Five of them were enriched in the mineral absorption pathway (*MT1E, MT2A, MT1X, HMOX1*, and *MT1F*); four were enriched in the worm longevity regulating pathway *(MT1E, MT2A, MT1X*, and *MT1F*), and two were enriched in pyrimidine metabolism (*NME1-NME2*, and *SMIM11A*).

### Transcription Factor Prediction From the DEGs

**Figure 5:**
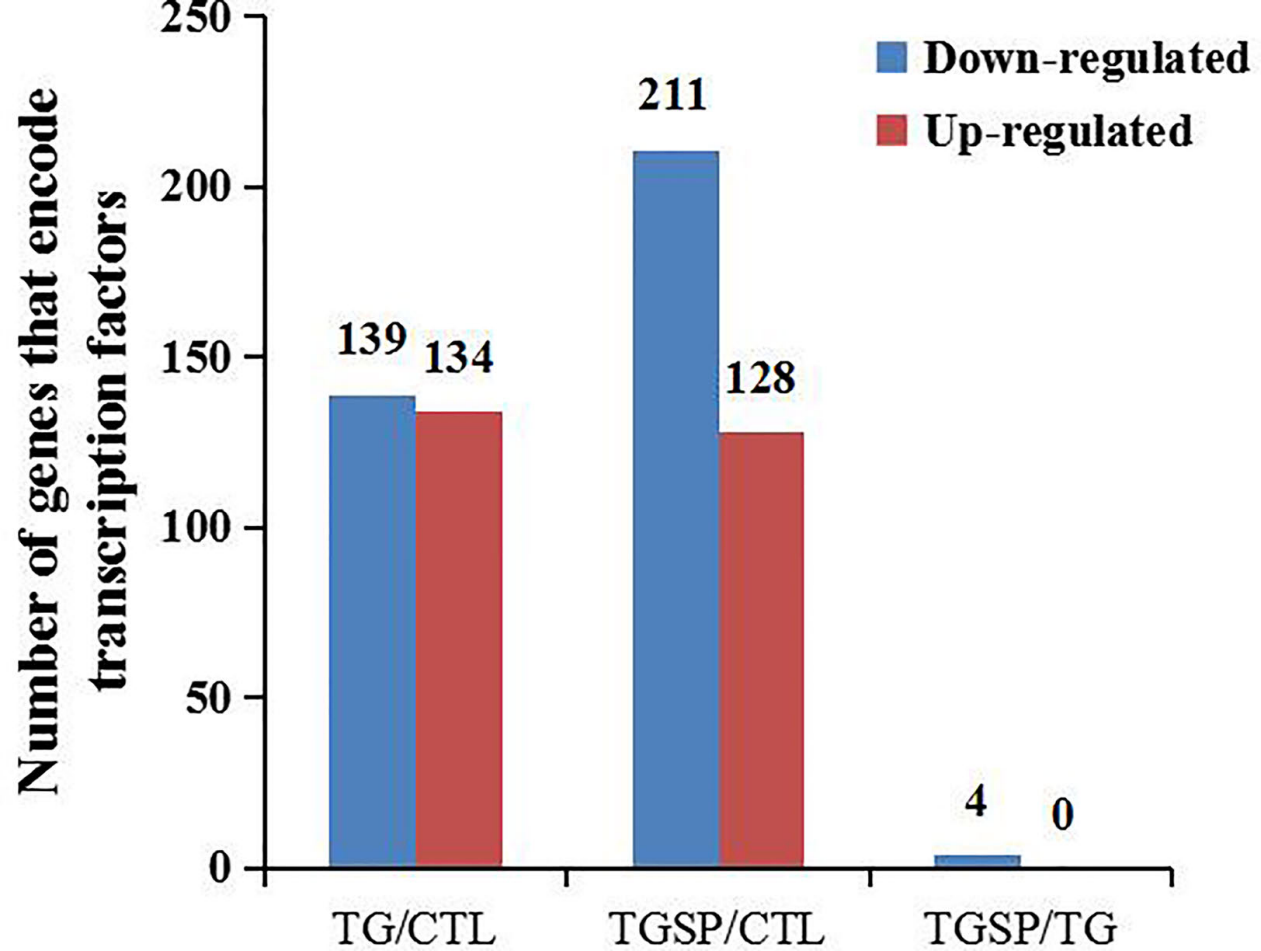
Transcription factor (TF) prediction. The number of TF-encoding DEGs changed more than 2-fold by the corresponding experimental treatment in each comparison pair.

We predicted the DEGs with the ability to encode transcription factors (TF). As shown in **Figure 5**, TG exposure induced the down-regulation of 139 TFs and more than 2-fold up-regulation of 134 TFs. In the TGSP group, the number of down-regulated TFs increased to 211 while the up-regulated TFs decreased to 128. However, compared to the TG group, no TF was up-regulated in the TGSP group, and only four down-regulated TFs were observed. Of the four down-regulated TFs, NM_058182.4 controls the transcription of *SMIM11A*, while NM_032827.6, XM_006712122.3, and XM_011533139.1 were responsible for the transcription of *ATOH8*, *ATOH8 transcript variant X4*, and *ATOH8 transcript variant X3*, respectively.

### Protein-Protein Interactions Among the DEGs

**Figure 6:**
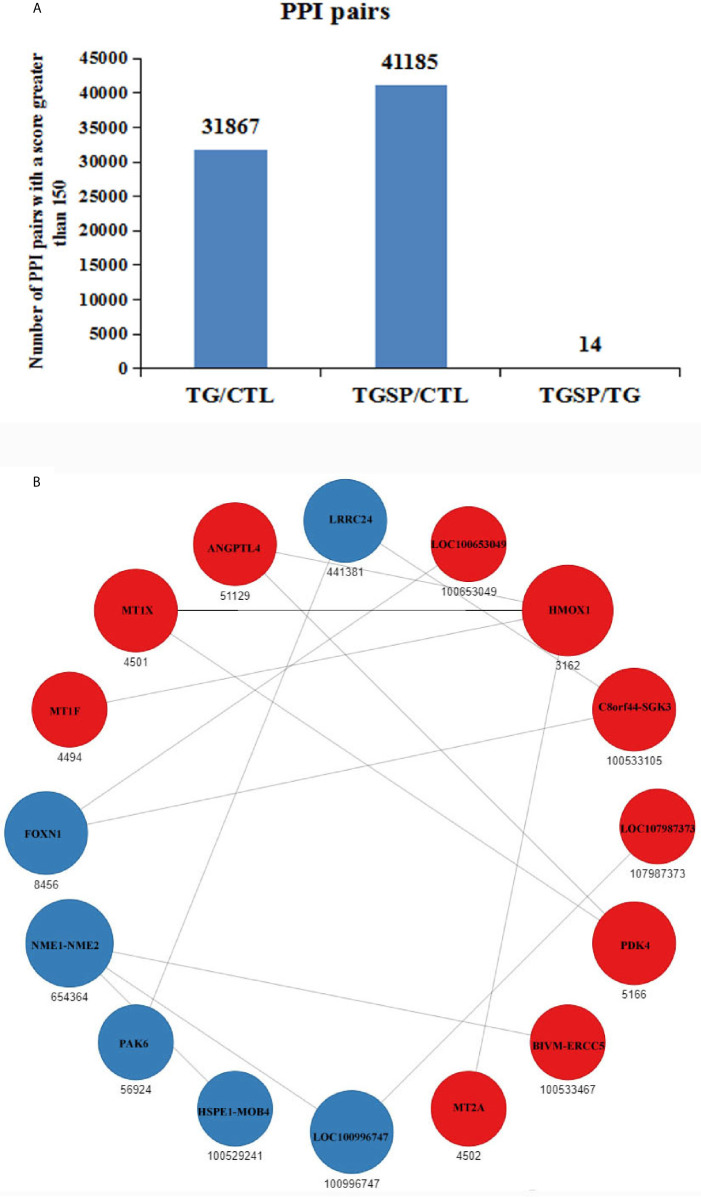
Prediction of the protein-protein interactions (PPI) for the DEGs. **(A)** The number of the predicted PPI pairs with a score greater than 150 according to the DEGs obtained from each comparison pair. **(B)** PPI network obtained from the TGSP *vs*.TG comparison pair. Red dots refer to up-regulated genes; blue dots refer to down-regulated genes. The number below the dots denotes the gene ID defined in the STRING database. The size of the circle indicates the number of interactions. The lines between dots represent the interactions.

Protein-protein interaction (PPI) networks among the DEG-encoded proteins were constructed using the homology with the known PPIs reported in the STRING database (24). As shown in **Figure 6A**, compared to the control group, the proteins encoded by the DEGs produced 31867 PPI pairs that had a score greater than 150 in the TG group. A high score indicated that the corresponding interaction had a high probability of occurring. Simultaneous sporamin treatment had increased the PPI pairs to 41185. However, when comparing the TGSP group to the TG group, only 14 PPI pairs were obtained from 16 DEGs. In this network, the expression of HMOX1, MT1F, MT1X, ANGPTL4, LOC100653049, C8orf44-SGK3, LOC107987373, PDK4, BIVM-ERCC5, and MT2A were up-regulated by sporamin treatment, while that of NME1-NME2, LRRC24, FOXN1, PAK6, HSPE1-MOB4, and LOC100996747 were down-regulated by sporamin. **Figure 6B** shows that HMOX1 and NME1-NME2 had more interactions (4 and 3, respectively) than the others where HMOX1 interacted with MT1X, MT1F, MT2A, and ANGPTL4 while NME1-NME2 interacted with BIVM-ERCC5, LOC100996747, and HSPE1-MOB4.

## Discussion

Sporamin has been shown to exert its anticancer roles in various cancer types such as CRC, pancreatic cancer, and tongue cancer. However, to date, its molecular mechanisms of action in different cancer cells remain poorly defined. RNA-seq is a powerful technique for the screening of the potential molecular targets of novel chemopreventive agents. Thus, in the present study, RNA-seq technology was applied to the *LoVo* human CRC cells after the treatment with sporamin in the presence of TG to look at the differences in the gene expression profiles of the cells after treatment, to obtain a comprehensive understanding of the effect of sporamin at the transcriptome level.

TG is a TPA type tumor migration promoter (13) that exerts its role as a non-competitive inhibitor of the sarco/endoplasmic reticulum Ca^2+^ ATPase, which discharges intracellular Ca^2+^ stores and causes a rapid and pronounced increase in the concentration of cytosolic free Ca^2+^ and acute responses in a large variety of cell types (28). It is also an epithelial anion secretagogue causing electrogenic anion secretion in monolayers of human colonic epithelial cells (29) and a histamine secretagogue inducing histidine decarboxylase activity and irritation of tissues (13). It has been shown to promote the migration of many CRC cell lines *in vitro* (30). Our study also demonstrated that TG promoted the migration of the LoVo cells in the wound healing assay. In contrast, sporamin inhibited the migration of the cells in the presence of TG.

In this study, sporamin treatment had brought some beneficial changes to the cells that were manifested as a gene expression profile favorable for the containment of an inflammatory or cancerous status of the cells. Compared to the TG-stimulated cells, sporamin treatment significantly up-regulated the mRNA expression of *RGPD2, SULT1A3*, and *BIVM-ERCC5*, but down-regulated that of *NPY4R, FOXN1, PAK6*, and *CEACAM20* as well as the transcription factors *SMIM11A* and *ATOH8*. Moreover, an interaction network formed by 16 proteins was attributable to sporamin treatment, where HMOX1 (up-regulated) and NME1-NME2 (down-regulated) seemed to be the primary nodes in that network.

The human *RGPD2* gene is also known as the *RANBP2*-like GRIP domain containing 2, which shares a high degree of sequence identity with mouse *RANBP2*, a sizeable nuclear pore protein that has been described to act as a tumor suppressor for its role in preventing chromosome segregation errors. Mice with decreased levels of this nucleoporin are highly sensitive to tumor formation (31). The *SULT1A3* gene encodes the phase II enzyme sulfotransferase 1A3/1A4 that catalyzes the sulfate conjugation of many hormones, neurotransmitters, drugs, and xenobiotic compounds (32) and are involved in both detoxification and bioactivation of various endogenous and exogenous compounds and are crucial for dramatically sensitizing the resistant cells to some anticancer agents (33). It also contributes to the bioavailability of dietary phenolic compounds (34) but its role in the utilization of sporamin has not been studied yet. The read-through transcript *BIVM-ERCC5* encodes a fusion protein that shares sequence identity with the products of the neighboring *BIVM* (basic, immunoglobulin-like variable motif containing) and *ERCC5* (excision repair cross-complementing rodent repair deficiency, complementation group 5) genes on chromosome 13. *BIVM* is a housekeeping gene but is poorly studied yet (35). The protein encoded by *ERCC5* is an endonuclease involved in the excision repair of UV-induced DNA damage (36). Mutational defects in *ERCC5* can cause either the cancer-prone condition xeroderma pigmentosum alone or in combination with the severe neurodevelopmental disorder Cockayne syndrome or the infantile lethal cerebro-oculo-facio-skeletal syndrome (37). An *ERCC5* mutant mouse model presented premature aging features, including cachexia and osteoporosis, with pronounced degenerative phenotypes in both liver and brain (37). On the contrary, some factors such as dietary restriction can substantially increase the lifespan of *ERCC5* mutant mice and delay aging, which has been attributed to the slowing of the accumulation of genome-wide DNA damage and preserving of the transcriptional output, thus contributing to improved cell viability (38). The aforementioned three genes were up-regulated by sporamin in the present study but the interactions between them still need further studies.

As to the four down-regulated DEGs, *NPY4R* is the gene for the neuropeptide Y receptor Y4 and is activated by the closely related peptide hormones neuropeptide Y (NPY), peptide YY and pancreatic polypeptide. NPY receptors are overexpressed in many cancers such as breast carcinomas and neuroblastomas (39) but their role in the development of CRC and the role of sporamin in the process are unknown. Carcinoembryonic antigen-related cell adhesion molecule (CEACAM) 20 is predominantly expressed in colonic epithelial cells and is an intestinal microvillus-specific transmembrane protein of the Ig superfamily. It is tyrosine-phosphorylated by the proto-oncogene c-Src (40) and consequently associated with the spleen tyrosine kinase (Syk) to form a complex, promoting the production of chemokines and cytokines in intestinal epithelial cells through activation of nuclear factor-κB (NF-κB), and then promoting the inflammatory conditions in the intestine (41). The *PAK6* gene is overexpressed in prostate cancer (42), hepatocellular carcinoma (43), cervical cancer (44), and colon cancer (45). And, its level has been correlated with the therapeutic resistance to 5-fluorouracil (45), docetaxel (46), and radiation (47). Moreover, down-regulating the expression of *PAK6* is an effective strategy in inhibiting the migration and invasion of colon cancer cells (48). The forkhead box N1 (*FOXN1*) gene belongs to the forkhead box gene family that comprises a diverse group of “winged-helix” transcription factors implicated in various biochemicals and cellular processes as development, metabolism, aging, and cancer. However, at present, the expression of *FOXN1* in the colon and the role of it in the development of CRC has not been studied yet. In the present study, these four genes were effectively down-regulated by sporamin, showing that they were possibly the molecular targets of sporamin in the *LoVo* colon cancer cells. If the effect can be further substantiated *in vivo*, it will be of greater importance. Subsequent GO enrichment analysis and KEGG pathway analysis confirmed that sporamin mainly increased the expression of the genes that contribute to the homeostasis of intracellular metal ions, the activities of essential enzymes, and the repairment of cellular DNA damages.

In the PPI network analysis, because interacting proteins tend to share similar functions (49), we only analyzed the significant nodes in the interaction network and found that heme oxygenase 1 (HMOX1) and NME1-NME2 were the primary nodes that had more interactions than other proteins. *HMOX1* is a human gene that encodes the enzyme heme oxygenase-1 (HO-1) that catalyzes the reaction that degrades the heme group contained in several important proteins such as hemoglobin, myoglobin, and cytochrome p450. Well-differentiated CRC seems to express more HO-1 than moderately/poorly-differentiated cancers so it is a useful diagnostic and prognostic indicator for CRC (50), and excessively increased expression of HO-1 in tumor cells may lead to cell death through a non-programmed cell death process called ferroptosis (51). The extract from *B. etnensis Raf.* has been shown to induce a ferroptotic cell death in human CRC cells by HO-1 hyper-expression (52). Sporamin had up-regulated *HMOX1* in the present study so it may also exert its role in this way, but more studies are needed to substantiate this hypothesis. The significance of the co-transcribed *NME1-NME2* mRNA and its predicted protein product’s function has not yet been determined. *NME1* and *NME2* have been reported to be over-expressed in CRC tumor tissues compared with normal tissues in a significant proportion of the patients (53). Sporamin down-regulated the expression of *NME1-NME2*, but to explicitly explain its effect on the fusion protein in the context of CRC, more work needs to be done.

In this study, two TFs, namely SMIM11A and ATOH8, were down-regulated by sporamin. However, we only know that SMIM11A, or small integral membrane protein 11A, is 58 amino acids long and is localized in the cytoplasm and mitochondria and focal adhesion points between the cells (*proteinatlas.org*). Its function, as well as its role in CRC, is poorly studied at present. Atonal homolog 8 (ATOH8) is a member of the basic-helix-loop-helix (bHLH) family of transcription factors and participates in embryogenesis and various tissues’ development. It is involved in the progression of malignancies too. Up-regulation of *ATOH8* promotes the intravascular survival of CRC cells in circulation (54), and a high expression of it predicts a poor clinical outcome in patients with CRC and contributes to tumor progression. Hence, it represents a potential prognostic predictor and therapeutic target in CRC (55). Therefore, it is important to study the interactions between sporamin and these two TFs in the future.

In conclusion, a comprehensive bioinformatic analysis of the transcriptomic profiles of the *LoVo* cells revealed that sporamin favorably altered the gene expression profile of the *LoVo* cancer cells in the presence of TG. It increased the expression of the genes that contribute to the homeostasis of intracellular metal ions, the activities of essential enzymes, and the repairment of cellular DNA damages. It may be helpful in the prevention and treatment of cancers but more focused *in vitro* and *in vivo* studies are needed to verify its effect on specific DEGs found in the present study and delineate the mechanism of action implicated in the process.

## Data Availability Statement

The original contributions presented in the study are publicly available. This data can be found here: https://www.ncbi.nlm.nih.gov/bioproject/PRJNA674402/.

## Author Contributions

P-GL and RX designed the research. S-JC and J-JZ conducted the experiment. CY analyzed data and wrote the manuscript. K-WZ and B-WC revised the manuscript. P-GL have primary responsibility for the final content. All authors agree to be accountable for the content of the work. All authors contributed to the article and approved the submitted version.

## Funding

This work was supported by the grants from the National Natural Science Foundation of China (No. 81573128 and 81703216).

## Conflict of Interest

The authors declare that the research was conducted in the absence of any commercial or financial relationships that could be construed as a potential conflict of interest.
