# Supplementary material for: Gene Expression Profile of the Human Colorectal Carcinoma LoVo Cells Treated With Sporamin and Thapsigargin
Source: Front Oncol. 2021 May 25;11:621462. doi: 10.3389/fonc.2021.621462 (PMC8185278; doi:10.3389/fonc.2021.621462)
Supplement: Supplementary file 1 [file Table_1.docx]

**Supplementary materials**

**Table S1.** Sequences of primers used in RT-qPCR

| Primers | Forward (5’-3’) | Reverse (5’-3’) |
| --- | --- | --- |
| GAPDH | GCACCGTCAAGGCTGAGAAC | TGGTGAAGACGCCAGTGGA |
| RGPD2 | GAGGACAGATGTTATTCAGGG | AGACACTTCAACTTCTGAAGC |
| SULT1A3 | GATCAGAAGGTCAAGGTGGT | TTCCATACGGTGGAAATGGT |
| BIVM-ERCC5 | TCAGAAGGAATGTCCTCAGC | ATGCAGTACCATCGTCTGAG |
| FOXN1 | GACTGACAGACGGACAGAG | GACACAAATGACGAGCAGC |
| PAK6 | TACAAGAGCTACCTGGTGG | CTCATTCAGCCTGACTTGG |
| CEACAM20 | GGTCCGAGTACTTGAAACAC | TCCACAGACCTAGCATTCTC |

**Table S2.** The numbers of the reads and their quality metrics for each sample

| Sample | Total Raw Reads  (Mb) | Total Clean Reads  (Mb) | Total Clean Bases  (Gb) | Clean Reads  Q20 (%) | Clean Reads  Q30 (%) | Clean Reads Ratio (%) |
| --- | --- | --- | --- | --- | --- | --- |
| CTL1 | 24.14 | 24.02 | 1.20 | 98.79 | 92.84 | 99.52 |
| CTL2 | 24.14 | 24.01 | 1.20 | 98.63 | 92.42 | 99.48 |
| CTL3 | 24.14 | 23.82 | 1.19 | 98.76 | 92.74 | 98.70 |
| TG1 | 24.14 | 24.06 | 1.20 | 98.67 | 92.58 | 99.69 |
| TG2 | 24.14 | 23.81 | 1.19 | 98.70 | 92.51 | 98.63 |
| TG3 | 24.14 | 23.58 | 1.18 | 98.69 | 92.71 | 97.69 |
| TGSP1 | 24.14 | 23.96 | 1.20 | 98.77 | 92.84 | 99.27 |
| TGSP2 | 24.14 | 23.98 | 1.20 | 98.73 | 92.62 | 99.34 |
| TGSP3 | 24.14 | 24.06 | 1.20 | 98.65 | 92.25 | 99.67 |

Notes: Samples: Sample names; Total Raw Reads (Mb): The reads amount before filtering, Unit: Mb; Total Clean Reads (Mb): The reads amount after filtering, Unit: Mb; Total Clean Bases (Gb): The total base amount after filtering, Unit: Gb; Clean Reads Q20 (%): The Q20 value for the clean reads; Clean Reads Q30 (%): The Q30 value for the clean reads; Clean Reads Ratio (%): The ratio of the amount of clean reads.

**Table S3.** Top 20 significantly down-regulated genes obtained from the TG vs. CTL comparison pair

| Genes | log_2_^Ratio TG/ CTL^ | Q-value | Modulation |
| --- | --- | --- | --- |
| *SULT1A3* | -8.01179 | 4.78E-28 | Down |
| *CT45A5* | -6.80433 | 3.36E-14 | Down |
| *HSD17B2* | -6.69356 | 2.43E-13 | Down |
| *C8orf44-SGK3* | -6.55306 | 2.52E-12 | Down |
| *AKR1C4* | -5.91909 | 1.21E-08 | Down |
| *LOC107986354* | -5.77851 | 1.61E-09 | Down |
| *SEMA5B* | -5.75015 | 7.15E-08 | Down |
| *C2orf71* | -5.29764 | 1.11E-10 | Down |
| *ALPI* | -5.1189 | 0 | Down |
| *CEACAM7* | -5.0941 | 1.98E-09 | Down |
| *CSH2* | -5.04971 | 2.13E-05 | Down |
| *LOC107987364* | -4.94792 | 4.05E-05 | Down |
| *CACNG4* | -4.85706 | 6.93E-05 | Down |
| *NT5C1B-RDH14* | -4.85087 | 7.19E-05 | Down |
| *MEOX1* | -4.72213 | 5.94E-72 | Down |
| *GPR20* | -4.72213 | 8.09E-37 | Down |
| *FOXN4* | -4.66862 | 5.17E-41 | Down |
| *MB* | -4.63467 | 0.00023 | Down |
| *MAGEC3* | -4.63347 | 3.91E-07 | Down |
| *LOC107985109* | -4.60798 | 0.000262 | Down |

**Table S4.** Top 20 significantly up-regulated genes obtained from the TG vs. CTL comparison pair

| Genes | log_2_^Ratio(TG/ CTL)^ | Q-value | Modulation |
| --- | --- | --- | --- |
| *NKX2-8* | 4.342609 | 3.76E-06 | Up |
| *COL26A1* | 4.369831 | 1.37E-70 | Up |
| *HIST1H2AA* | 4.409723 | 2.11E-06 | Up |
| *SETDB2-PHF11* | 4.438512 | 1.04E-43 | Up |
| *NT5C1B* | 4.535254 | 0.000302 | Up |
| *PRPH* | 4.676055 | 1.67E-13 | Up |
| *GOLGA8R* | 4.678212 | 0.000146 | Up |
| *CYP4F2* | 4.723033 | 1.03E-08 | Up |
| *DACT3* | 4.904488 | 1.21E-08 | Up |
| *BHLHA15* | 5.001954 | 1.30E-91 | Up |
| *GOLGA6L1* | 5.05798 | 1.48E-05 | Up |
| *KRT33B* | 5.203525 | 5.28E-06 | Up |
| *AMY1C* | 5.260632 | 3.44E-06 | Up |
| *DPYSL3* | 5.473854 | 2.94E-12 | Up |
| *RIMBP3C* | 5.885752 | 1.00E-08 | Up |
| *LOC100996318* | 6.120217 | 6.02E-10 | Up |
| *LILRB3* | 6.484789 | 3.23E-12 | Up |
| *KLHDC7B* | 6.783182 | 0 | Up |
| *DHRS2* | 7.247764 | 0 | Up |
| *BIVM-ERCC5* | 7.757003 | 4.21E-25 | Up |

**Table S5.** Top 20 significantly down-regulated genes obtained from the TGSP vs. CTL comparison pair

| Symbol | log_2_^Ratio(TGSP / CTL)^ | Q-value | Modulation |
| --- | --- | --- | --- |
| *RPL17-C18orf32* | -6.43944 | 4.39E-13 | Down |
| *HSD17B2* | -6.34075 | 2.24E-12 | Down |
| *MAGEC2* | -6.00323 | 1.26E-18 | Down |
| *MEOX1* | -5.95428 | 1.03E-66 | Down |
| *VHLL* | -5.90244 | 9.78E-06 | Down |
| *LOC100996747* | -5.88514 | 1.37E-09 | Down |
| *AKR1C4* | -5.56627 | 4.85E-08 | Down |
| *LGALS9C* | -5.49183 | 1.01E-07 | Down |
| *ALPI* | -5.48885 | 0 | Down |
| *MYCN* | -5.45178 | 1.48E-07 | Down |
| *NCALD* | -5.34075 | 2.07E-12 | Down |
| *LOC102723730* | -5.28186 | 6.81E-07 | Down |
| *MAGEC3* | -5.28065 | 6.88E-07 | Down |
| *CHRNA4* | -5.15633 | 1.91E-06 | Down |
| *FOXN4* | -5.12316 | 7.34E-38 | Down |
| *PRDM16* | -4.9823 | 5.77E-10 | Down |
| *LOC107985773* | -4.94558 | 9.10E-06 | Down |
| *C2orf71* | -4.94482 | 9.73E-10 | Down |
| *NPY4R* | -4.91414 | 0 | Down |
| *HPX* | -4.78436 | 2.62E-05 | Down |

**Table S6.** Top 20 significantly up-regulated genes obtained from the TGSP vs CTL comparison pair

| Symbol | log_2_^Ratio(TGSP / CTL)^ | Q-value | Modulation |
| --- | --- | --- | --- |
| *NGFR* | 3.922016 | 1.35E-21 | Up |
| *NKX2-8* | 4.003546 | 7.87E-05 | Up |
| *MAP2* | 4.209997 | 3.21E-16 | Up |
| *BHLHE41* | 4.2927 | 5.56E-71 | Up |
| *BHLHA15* | 4.404645 | 2.69E-52 | Up |
| *SLC30A2* | 4.473032 | 0.000524 | Up |
| *TMC3* | 4.473032 | 0.000524 | Up |
| *TBC1D3H* | 4.579603 | 0.00033 | Up |
| *SETDB2-PHF11* | 4.675132 | 6.78E-47 | Up |
| *PRPH* | 4.942385 | 9.59E-15 | Up |
| *GOLGA8R* | 5.148095 | 1.56E-05 | Up |
| *GOLGA6L1* | 5.222447 | 9.55E-06 | Up |
| *KRT33B* | 5.235735 | 8.73E-06 | Up |
| *DACT3* | 5.347501 | 1.89E-10 | Up |
| *DPYSL3* | 5.551034 | 8.52E-12 | Up |
| *GSTT2* | 5.582578 | 6.43E-07 | Up |
| *LILRB3* | 6.865349 | 7.64E-14 | Up |
| *DHRS2* | 6.917578 | 0 | Up |
| *KLHDC7B* | 6.917816 | 0 | Up |
| *BIVM-ERCC5* | 8.880611 | 3.70E-42 | Up |

**Table S7.** Top 20 significantly down-regulated genes obtained from the TGSP vs TG comparison pair

| Symbol | log_2_^Ratio(TGSP / TG)^ | Q-value | Modulation |
| --- | --- | --- | --- |
| *LOC100996747* | -6.31961 | 5.94E-11 | Down |
| *RIMBP3C* | -5.58265 | 6.32E-07 | Down |
| *AMY1C* | -4.95753 | 9.78E-05 | Down |
| *U2AF1L5* | -3.94164 | 2.99E-10 | Down |
| *LOC100996318* | -3.5126 | 1.20E-06 | Down |
| *PAK6* | -2.4866 | 1.96E-10 | Down |
| *HSPE1-MOB4* | -2.26758 | 7.91E-11 | Down |
| *SMIM11A* | -2.08986 | 3.15E-09 | Down |
| *ATOH8* | -2.06222 | 0.00078 | Down |
| *LOC105376906* | -1.96681 | 0.0004 | Down |
| *TNFSF12-*  *TNFSF13* | -1.65687 | 7.33E-07 | Down |
| *OR2B6* | -1.55002 | 0.000238 | Down |
| *NPY4R* | -1.3945 | 2.14E-05 | Down |
| *LOC107987425* | -1.3808 | 7.43E-17 | Down |
| *CEACAM20* | -1.2225 | 2.84E-07 | Down |
| *NME1-NME2* | -1.21788 | 3.31E-13 | Down |
| *FOXN1* | -1.19973 | 0.000145 | Down |
| *LOC102723360* | -1.15461 | 6.71E-06 | Down |
| *ST20-MTHFS* | -1.14058 | 3.47E-10 | Down |
| *OLFML1* | -1.05047 | 2.37E-12 | Down |

**Table S8.** Top 20 significantly up-regulated genes obtained from the TGSP vs. TG comparison pair

| Symbol | log_2_^Ratio(TGSP / TG)^ | Q-value | Modulation |
| --- | --- | --- | --- |
| *RAB3A* | 1.215311 | 2.63E-06 | Up |
| *HMOX1* | 1.228805 | 3.92E-46 | Up |
| *MT2A* | 1.2604 | 5.53E-98 | Up |
| *MT1E* | 1.581501 | 0 | Up |
| *RGPD2* | 1.599449 | 0.000186 | Up |
| *ANGPTL4* | 1.659764 | 1.26E-96 | Up |
| *NPIPB3* | 1.673435 | 1.38E-49 | Up |
| *KIR2DL5B* | 1.686669 | 1.74E-16 | Up |
| *ZHX1-C8orf76* | 1.731097 | 1.60E-05 | Up |
| *CEMP1* | 1.822001 | 7.29E-08 | Up |
| *FSBP* | 1.851642 | 7.85E-07 | Up |
| *LOC100653049* | 2.939799 | 2.18E-08 | Up |
| *MT1X* | 3.209639 | 1.77E-57 | Up |
| *MT1F* | 3.355426 | 0.000354 | Up |
| *LOC107987364* | 5.187728 | 0.000183 | Up |
| *C8orf44-SGK3* | 5.393707 | 4.97E-05 | Up |
| *LOC107987373* | 5.460503 | 3.17E-05 | Up |
| *CT45A5* | 6.479209 | 1.25E-09 | Up |
| *LOC107986354* | 6.768488 | 4.75E-14 | Up |
| *SULT1A3* | 9.105496 | 4.61E-45 | Up |

**Table S9.** Enriched gene ontology (GO) terms with a *P-value* <0.05 for the DEGs obtained from the TG vs. CTL comparison pair

|  | **Terms** | **Number of DEGs** | ***P-value*** |
| --- | --- | --- | --- |
| CC | endoplasmic reticulum | 204 | 2.62E-08 |
|  | extracellular region part | 395 | 4.62E-08 |
|  | extracellular region | 443 | 2.38E-06 |
|  | extracellular space | 366 | 5.68E-06 |
|  | extracellular vesicle | 294 | 1.19E-05 |
|  | extracellular organelle | 294 | 1.23E-05 |
|  | endoplasmic reticulum part | 145 | 3.73E-05 |
|  | extracellular exosome | 289 | 5.46E-05 |
|  | endoplasmic reticulum chaperone complex | 8 | 0.0001 |
|  | endomembrane system | 381 | 0.00018 |
|  | vesicle | 382 | 0.00122 |
|  | organelle subcompartment | 175 | 0.00209 |
|  | extracellular matrix | 63 | 0.0022 |
|  | endoplasmic reticulum lumen | 36 | 0.00242 |
|  | nuclear outer membrane-endoplasmic reticulum membrane network | 121 | 0.0031 |
|  | endoplasmic reticulum membrane | 119 | 0.00324 |
|  | endoplasmic reticulum subcompartment | 119 | 0.00339 |
|  | nucleosome | 20 | 0.00595 |
|  | proteinaceous extracellular matrix | 50 | 0.00896 |
|  | DNA packaging complex | 20 | 0.01987 |
|  | intrinsic component of endoplasmic reticulum membrane | 26 | 0.02103 |
|  | integral component of endoplasmic reticulum membrane | 25 | 0.03102 |
| BP | response to unfolded protein | 47 | 3.96E-15 |
|  | response to topologically incorrect protein | 47 | 2.95E-14 |
|  | cellular response to unfolded protein | 35 | 1.33E-12 |
|  | endoplasmic reticulum unfolded protein response | 34 | 6.68E-12 |
|  | cellular response to topologically incorrect protein | 35 | 6.70E-12 |
|  | response to endoplasmic reticulum stress | 42 | 6.27E-11 |
|  | activation of signaling protein activity involved in unfolded protein response | 27 | 3.71E-09 |
|  | positive regulation of nuclease activity | 27 | 8.32E-09 |
|  | regulation of nuclease activity | 28 | 1.17E-08 |
|  | negative regulation of coagulation | 13 | 0.01738 |
|  | negative regulation of wound healing | 13 | 0.02415 |
|  | negative regulation of blood coagulation | 12 | 0.02789 |
|  | negative regulation of hemostasis | 12 | 0.02789 |
|  | positive regulation of hydrolase activity | 56 | 0.04109 |
| MF | gated channel activity | 48 | 0.00655 |
|  | substrate-specific channel activity | 60 | 0.00756 |
|  | ion channel activity | 58 | 0.0098 |
|  | androsterone dehydrogenase activity | 4 | 0.0385 |
|  | channel activity | 61 | 0.03991 |
|  | passive transmembrane transporter activity | 61 | 0.04262 |

Notes: CC: cellular component; BP: biological process; MF: molecular function

**Table S10.** Enriched GO terms with a *P-value* <0.05 for the DEGs obtained from the TGSP vs. CTL comparison pair

|  | **term** | **Number of DEGs** | ***P-value*** |
| --- | --- | --- | --- |
| CC | endoplasmic reticulum | 228 | 7.03E-08 |
|  | endomembrane system | 443 | 1.48E-05 |
|  | extracellular region part | 432 | 2.78E-05 |
|  | extracellular region | 491 | 0.00027 |
|  | endoplasmic reticulum chaperone complex | 8 | 0.00036 |
|  | extracellular space | 402 | 0.00084 |
|  | endoplasmic reticulum part | 157 | 0.00086 |
|  | organelle subcompartment | 199 | 0.00181 |
|  | vesicle | 434 | 0.00292 |
|  | extracellular vesicle | 317 | 0.00473 |
|  | extracellular organelle | 317 | 0.00488 |
|  | plasma membrane part | 295 | 0.00915 |
|  | endoplasmic reticulum-Golgi intermediate compartment | 25 | 0.00932 |
|  | extracellular exosome | 313 | 0.01072 |
|  | nuclear outer membrane-endoplasmic reticulum membrane network | 134 | 0.01078 |
|  | intrinsic component of plasma membrane | 200 | 0.01537 |
|  | endoplasmic reticulum membrane | 131 | 0.01553 |
|  | endoplasmic reticulum subcompartment | 131 | 0.01628 |
|  | Golgi apparatus | 178 | 0.02977 |
|  | integral component of plasma membrane | 192 | 0.02991 |
| BP | response to unfolded protein | 50 | 7.84E-15 |
|  | response to topologically incorrect protein | 50 | 6.59E-14 |
|  | endoplasmic reticulum unfolded protein response | 38 | 2.34E-13 |
|  | cellular response to unfolded protein | 38 | 3.72E-13 |
|  | response to endoplasmic reticulum stress | 48 | 7.12E-13 |
|  | cellular response to topologically incorrect protein | 38 | 2.19E-12 |
|  | activation of signaling protein activity involved in unfolded protein response | 31 | 4.54E-11 |
|  | positive regulation of nuclease activity | 31 | 1.20E-10 |
|  | regulation of nuclease activity | 32 | 2.51E-10 |
|  | response to acid chemical | 49 | 0.00112 |
|  | positive regulation of hydrolase activity | 65 | 0.00704 |
|  | regulation of hydrolase activity | 95 | 0.01549 |
|  | ubiquitin-dependent ERAD pathway | 13 | 0.02294 |
|  | protein N-linked glycosylation | 24 | 0.02378 |
|  | cell communication | 420 | 0.02646 |
|  | cellular response to acid chemical | 28 | 0.02932 |
|  | neutral lipid biosynthetic process | 15 | 0.03207 |
|  | acylglycerol biosynthetic process | 15 | 0.03207 |
|  | ERAD pathway | 13 | 0.03338 |
| MF | substrate-specific channel activity | 71 | 0.00041 |
|  | gated channel activity | 56 | 0.00074 |
|  | ion channel activity | 68 | 0.00091 |
|  | channel activity | 73 | 0.00185 |
|  | passive transmembrane transporter activity | 73 | 0.00201 |

Notes: CC: cellular component; BP: biological process; MF: molecular function

**Table S11.** Enriched GO terms with a *P-value* <0.05 for the DEGs obtained from the TGSP vs. TG comparison pair

|  | **term** | **Number of DEGs** | ***P-value*** |
| --- | --- | --- | --- |
| CC |  | 0 |  |
| BP | cellular response to zinc ion | 4 | 7.84E-07 |
|  | cellular response to cadmium ion | 4 | 1.06E-06 |
|  | cellular response to metal ion | 5 | 2.39E-05 |
|  | cellular response to inorganic substance | 5 | 3.04E-05 |
|  | response to cadmium ion | 4 | 4.02E-05 |
|  | response to zinc ion | 4 | 5.65E-05 |
|  | response to metal ion | 5 | 0.004 |
|  | response to erythropoietin | 2 | 0.00981 |
|  | cellular response to erythropoietin | 2 | 0.00981 |
|  | response to inorganic substance | 5 | 0.01539 |
| MF |  | 0 |  |

Notes: CC: cellular component; BP: biological process; MF: molecular function
